# Early potential metabolic biomarkers of T1 stage lung adenocarcinoma based on serum metabolomics

**DOI:** 10.3389/fmolb.2025.1544774

**Published:** 2025-04-25

**Authors:** Bin Wu, Min Zheng, Qingkui Guo, Ning Wang, Chen Zhu, Wen Zhao, Ye Xu

**Affiliations:** Department of Thoracic Surgery, Tongren Hospital, Shanghai Jiaotong University School of Medicine, Shanghai, China

**Keywords:** lung adenocarcinoma, biomarker, metabolomics, lipid metabolism, LC-MS

## Abstract

**Background:**

This study aims to investigate serum metabolite changes in patients with early-stage (T1) lung adenocarcinoma, identify potential diagnostic biomarkers, and establish an early warning mechanism for T1 stage lung adenocarcinoma.

**Methods:**

The study included two groups: a lung adenocarcinoma group and a healthy control group. Serum samples underwent non-targeted metabolomics analysis. Total ion chromatograms (TIC) were generated to assess system stability. Chromatographic data were analyzed using multivariate statistical methods, including principal component analysis (PCA) for dimensionality reduction. Partial least squares discriminant analysis (PLS-DA) further validated PCA findings. Variables with VIP scores >1.0 in the PLS-DA model were selected, combined with ANOVA and T-tests (P < 0.05), to identify differentially expressed metabolites. Receiver operating characteristic (ROC) curve analysis was conducted to evaluate the diagnostic performance of selected metabolites.

**Results:**

Serum metabolites significantly differed between the lung adenocarcinoma group and the healthy control group. Multivariate statistical analysis and ROC curve evaluation identified four potential diagnostic biomarkers: Cortisol, 3-Oxo-OPC4-CoA, PE-NMe(14:1(9Z)/14:1(9Z)), and Ceramide (d18:1/9Z-18:1), with AUC values of 0.930, 0.895, 0.890, and 0.795, respectively.

**Conclusion:**

Cortisol,3-Oxo-OPC4-CoA,PE-NMe(14:1(9Z)/14:1(9Z)), and Ceramide (d18:1/9Z-18:1) exhibit significantly altered metabolic levels in T1 stage lung adenocarcinoma patients and can serve as metabolic biomarkers. These markers may enhance the sensitivity and specificity of early diagnosis, facilitating improved detection of T1 stage lung adenocarcinoma.

## Background

Lung cancer is one of the most common malignant tumors globally, with the highest morbidity and mortality (Adams et al., 2023). In the past few years, the incidence and death rate of lung cancer in China has increased. According to the global cancer statistics for 2020 reported by the International Agency for Research on Cancer, there will be approximately 820,000 new lung cancer diagnoses and 715,000 lung cancer-related deaths in China in 2020 (Sung et al., 2021; Cao et al., 2021). Due to the latent clinical symptoms of the diseases in the early stages and the lack of effective treatment options for advanced disease, lung cancer is associated with a high mortality rate. Lung cancer has a variety of subtypes, and in addition to morphological and histological differences, different subtypes also have different tumor progression patterns. Lung cancer can be divided into small-cell lung cancer (15%) and non-small cell lung cancer (85%) according to the pathological morphological characteristics and differentiation of cells (Buma et al., 2023). Non-small cell lung cancer (NSCLC) includes adenocarcinoma, squamous cell carcinoma and large cell carcinoma, accounting for about 50%, 35% and 15%, respectively. The most common histological subtype of NSCLC is adenocarcinoma, followed by squamous cell carcinoma, and the proportion of histological subtypes varies by ethnicity. Among all NSCLC subtypes, lung adenocarcinoma accounts for about 47% of Western patients and about 55%–60% in Chinese patients (Chen et al., 2022). In daily clinical practice, imaging and clinical symptoms can no longer reflect the full disease status of patients. Studies indicate that a majority of lung cancer patients are diagnosed at advanced stages (stage III/IV) or with metastatic disease, facing a poor prognosis characterized by five-year survival rates as low as 7% for metastatic NSCLC and 3% for SCLC, alongside limited curative treatment options beyond systemic therapies such as chemotherapy or targeted agents (Hirsch et al., 2017; Blandin Knight et al., 2017; Li et al., 2018). Because tumor marker measurement can provide a rapid, minimally invasive, and safe real-time assessment of a patient’s tumor status, it can be used as an early diagnostic tool for lung cancer. Therefore, searching for early warning markers of lung cancer can improve the early detection rate of lung cancer patients and improve the clinical outcome of lung cancer.

In recent years, the important role of changes in cell metabolism in the development of tumors has been gradually recognized. During early tumorigenesis, when alterations in protein abundance or gene expression remain undetectable by conventional methods, metabolomic shifts-driven by both endogenous dysregulation (e.g., oncogene-induced enzymatic activity changes) and external stimuli (e.g., microenvironmental interactions)-can be identified through sensitive analytical platforms such as mass spectrometry. Unlike proteomics or genomics, where sensitivity primarily refers to detecting low-abundance molecules (e.g., cancer-related proteins at pg/mL levels), the unique advantage of metabolomics lies in its ability to capture early functional perturbations in biochemical pathways. These metabolic alterations often precede measurable changes in protein/gene expression, as small-molecule metabolites (e.g., lactate, 2-hydroxyglutarate) rapidly accumulate or deplete in response to nascent tumorigenic signals, enabling earlier detection of pathological states before morphological tumor formation (Patti et al., 2012; Mayers et al., 2014). Metabolomics is a rapid method for qualitative and semi-quantitative analysis of metabolites in cells, biological fluids, and tissues. As a relatively new tool for disease biomarker identification, metabolomics has been applied to identify early diagnostic markers of various diseases (Chen et al., 2019). With the continuous recognition of the important role of metabolomics in the early diagnosis of tumors, lipid molecules, as an essential class of metabolites in the human body, have a variety of critical biological functions, such as biofilm composition, vesicle transport, adhesion, migration, apoptosis, energy storage, neurotransmission, signal transduction, and post-translational modification (Zhu et al., 2020). According to the International Committee on the Classification and Nomenclature of Lipids, lipids are mainly divided into the following types: fatty acids, glycerolipids, glycerophospholipids, sphingolipids, sterol lipids, prenol lipids, saccharolipids, and polyketides. At present, fatty acids, glycerolipids, ,sphingomyelins and sterol lipids are considered most relevant to tumor development and chemotherapy (Huang and Freter, 2015). With the deepening of the study of lipid metabolism, lipid metabolites have gradually developed into metabolomics, which is a more systematic study of the occurrence and development of tumors.

Metabolomics, as a system biological technique for the large-scale determination of metabolites, has been applied to the study of various human diseases. Given the systematic characteristics of the omics method, applying metabolomics can generate a complete metabolic landscape map and conduct a comprehensive analysis to identify key metabolic factors in disease pathology (Lin et al., 2021). At present, the primary detection methods include nuclear magnetic resonance (NMR), liquid-mass coupling (LC-MS), gas-mass coupling (GC-MS) and chromatography, etc. Untargeted metabolomics enables the discovery of disease-specific biomarkers by analyzing metabolic profiles through high-resolution spectral detection and chemical pattern recognition. To ensure biological interpretability, this approach requires a tightly defined research question-such as identifying cancer-associated metabolic perturbations-rather than conflating distinct objectives like drug efficacy and toxicity assessment. By focusing on cohort-stratified metabolic signatures (e.g., tumor vs. normal tissue) and validating candidates via orthogonal assays, metabolomics offers a robust framework for biomarker identification, as demonstrated in recent studies linking dysregulated pathways (e.g., glycolysis, nucleotide metabolism) to cancer progression (Wishart, 2016; Johnson et al., 2016). Studies have shown that the disturbance of lipid metabolism can change the composition and permeability of cell membranes, leading to the occurrence and development of various tumors, and abnormal lipid metabolism has been found in colon cancer, breast cancer, prostate cancer and other tumors (Long et al., 2018). However, there are few effective reports on the early diagnostic markers of T1 lung adenocarcinoma based on lipid metabolites. Currently, common biomarkers for lung cancer include CYFRA 21-1, carcinoembryonic antigen, neuron-specific enolase, and squamous cell carcinoma antigen (Nooreldeen and Bach, 2021). These biological criteria have shown poor diagnostic value in daily clinical practice and are unsuitable for early detection of T1 lung adenocarcinoma. In addition, it is difficult to ac accurately diagnose and classify a disease with a single marker, and the combination of multiple markers can effectively improve the sensitivity and specificity of diagnosis.

In summary, current clinical practice for T1 lung adenocarcinoma lacks reliable early detection biomarkers, underscoring the urgent need to identify and validate highly sensitive diagnostic markers to address this critical gap in early-stage disease management. Our study employed untargeted metabolomic profiling of serum samples from lung adenocarcinoma patients and healthy controls to identify biomarkers with robust diagnostic potential, establishing a foundation for early detection of stage T1 lung adenocarcinoma.

## Material and methods

### Chemical and instrumentation

LC/MS grade acetonitrile and HPLC grade methanol were purchased from Merck (Dannstadt, Germany), formic acid and L-2-chloro-phenylalanine were purchased from Sigma-Aldrich (St. Louis, MO, USA), and the experimental water was Watsons’ distilled water. All other reagents were commercially analytically pure. The Thermo Fisher FRESCO17 cryogenic centrifuge is used for ultra-high speed centrifugation of samples.

### Patients

This study enrolled 20 treatment-naïve patients with surgically confirmed stage T1 lung adenocarcinoma (tumor size: 1–3 cm) and 20 age-matched healthy controls (mean age: 61.96 vs. 62.76 years) from Shanghai Jiao Tong University Affiliated Tongren Hospital (2021-2022). All patients exhibited upper lobe predominance (right upper: 7; left upper: 8) with standardized nodule sizes to minimize disease heterogeneity, while controls had no cancer history and matched BMI (18.5-23.9). Exclusion criteria included metabolic disorders (obesity, diabetes, hyperlipidemia), concurrent medications, or systemic diseases. Demographic details (age, sex distribution: patients 6F/14M vs. controls 10F/10M) and tumor localization are summarized in Table 1. The study protocol (IRB: 2021-080-02) was approved by the hospital ethics committee, with written informed consent obtained from all participants.

**TABLE 1:** The characteristics of the selected population.

Factors		Lung cancer (n = 20)	Health (n = 20)
Age (years)	Mean	61.96	62.76
Sex	Female/male	6/14	10/10
Location	Right upper	7	
	Right middle	1	
	Right lower	2	
	Left upper	8	
	Left lower	2	

### Samples collection and preparation

All blood samples were taken from subjects before fasting to avoid the influence of diet on lipid metabolites in blood. Venous blood was placed at 25°C for 10 min and centrifuged at 2,000 rpm for 20 min. About 100 μL serum was added to 300 μL methanol solution containing 5 μg/mL L-2-chlorophenylalanine as the internal standard and rotated for 2 min. Centrifuge at 13,000 rpm for 10 min at 4°C and obtain 200 μL supernatant. The same volume of serum is extracted from all samples and mixed evenly to prepare quality control samples.

### Chromatographic condition

In this study, ACQUITYTM UPLC-Waters Xevo G2-XS QTof high resolution mass spectrometry system was used. For chromatographic separation, a Waters HSS T3 analytical column of 2.1 × 100 mm and 1.8 μM was employed, along with a Waters HSS T3 guard column. The sample size was 2 μL, the column temperature was 25°C, the flow rate was 0.4 mL/min.The elution protocol was initiated at a flow rate of 0.4 mL/min with 98% A and 5% B (0–3 min), followed by a linear gradient to 2% A and 98% B over 13 min (3–16 min), and maintained at 98% B for an additional 2 min (16–18 min) to ensure complete elution of analytes as shown in Table 2. The total runtime of the method was 18 min under consistent flow rate conditions. Data acquisition and processing were conducted using the instrument-integrated MassLynx™ software (v4.2).

**TABLE 2:** Elution gradient of the mobile phase.

Time (min)	Flow rate (mL/min)	A (%)	B (%)
0	0.4	98	5
3	0.4	98	5
16	0.4	2	98
18	0.4	2	98

### Mass spectrum conditions

The mass spectrometer operated in both positive and negative electrospray ionization (ESI) modes, with capillary voltages set to 2.5 kV (ESI^+^) and 2.0 kV (ESI^−^), a cone voltage of 40 V, and a mass acquisition range of m/z 50-1500 to cover small-molecule metabolites. High-resolution data were acquired at a scan rate of 0.2 s/scan with a resolution exceeding 20,000 FWHM. Ion source conditions included a temperature of 120°C, desolvation gas (N_2_) flow of 800 L/h at 500°C, and cone gas flow of 50 L/h. For structural elucidation, data-dependent acquisition (DDA) was employed with dynamic collision energy (15–40 eV) to fragment precursor ions. Real-time mass calibration utilized leucine enkephalin (m/z 556.2771 for ESI^+^ or 554.2615 for ESI^−^) via LockSpray every 30 s, ensuring mass accuracy below 5 ppm. System stability was monitored using daily QC samples, with retention time drift <0.1 min and peak intensity RSD <15%.

### Data processing and statistical analysis

Raw data acquired in both positive and negative ionization modes (*m/z* 50–1500) were imported into Progenesis QI™ (v3.0) for preprocessing, including noise reduction, baseline correction, and peak alignment across all samples. Feature detection was conducted with a sensitivity threshold of 10 counts and a minimum peak width of 0.1 min, while isotopic and adduct ion clusters were deconvoluted to generate a refined list of molecular features. Quality control (QC) samples, comprising pooled aliquots of all study samples, were analyzed to monitor technical variability, with features exhibiting >15% relative standard deviation (RSD) in QCs excluded from downstream analysis. Metabolite identification was performed using Progenesis QI™ software (version 3.0, Waters Corporation) with optimized parameters to ensure robust and reproducible analysis. Raw LC-HRMS data files (.raw) were imported into the software for automated peak detection, alignment, and deconvolution, applying a signal-to-noise ratio (S/N) threshold of 10 and a minimum peak width of 0.1 min to distinguish true metabolic features from background noise. Retention time alignment across all samples was conducted with a tolerance of 0.2 min to correct for instrumental drift, while isotopic and adduct ion clusters (e.g., [M+H]^+^, [M+Na]^+^, [M−H]^−^) were resolved using a mass error tolerance of <5 ppm and an intensity threshold of 1,000 counts. Feature quantification was based on area-under-the-curve (AUC) integration, with baseline subtraction performed dynamically to minimize noise interference. Metabolite annotation was achieved by matching accurate mass (<5 ppm error) and MS/MS fragmentation patterns (when available) against the Human Metabolome Database (HMDB), METLIN, and an in-house library of authenticated standards. For unidentified features, tentative annotations were assigned using elemental composition prediction and neutral loss analysis. Final data were normalized to internal standards and exported for visualization in tools such as SIMCA-P+ 13.0 software for multivariate statistical analysis, and total ion chromatogram (TIC) was established to investigate the stability of the instrument. Principal component analysis (PCA) and partial least squares-discriminant analysis (PLS-DA), were applied to identify cohort-specific metabolic signatures.For differential metabolite screening, a dual statistical approach combining univariate and multivariate analyses was applied: univariate analysis identified features with significant fold changes and adjusted p-values (FDR <0.05 via Benjamini-Hochberg correction), while multivariate analysis using partial least squares-discriminant analysis (PLS-DA) prioritized metabolites based on variable importance in projection (VIP scores >1.0). Metabolites meeting both criteria (FC > 1.2 or FC < 0.6, FDR <0.05, and VIP >1.0) were classified as high-confidence differential candidates. Then, the area under the ROC curve was used to evaluate the diagnostic ability of differential metabolites. Finally, bioinformatics analysis was performed by Ingenuity Pathway Analysis (IPA) software to identify the differential metabolite interaction networks. IPA is a robust bioinformatics tool designed to interpret complex biological data by integrating experimental findings with curated knowledge from scientific literature and databases. In the context of metabolomics, IPA facilitates the construction of metabolite interaction networks through a systematic workflow. Initially, differentially expressed metabolites identified via untargeted metabolomics are uploaded into the IPA platform, accompanied by identifiers such as HMDB or KEGG IDs to ensure accurate annotation. The software maps these metabolites to its Knowledge Base, which encompasses manually curated interactions, pathways, and functional associations derived from peer-reviewed studies.

## Results

### Multivariate statistical analysis

**FIGURE 1:**
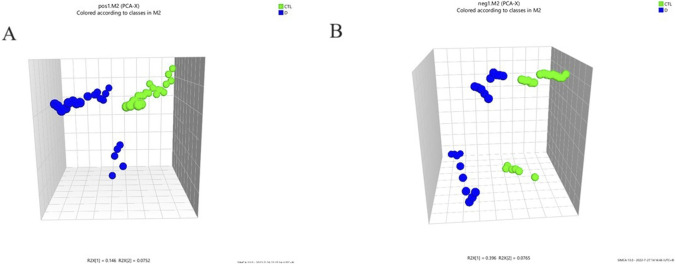
Principle component analysis (PCA) 3D scores plot to discriminate the metabolic profiles in serum of the lung adenocarcinoma group and healthy control group. POS (two components model: R2X[1] = 0.146, R2X[2] = 0.0752).NEG (two components model: R2X[1] = 0.396, R2X[2] = 0.0765); **(A)** PCA score of the two groups (pos); **(B)** PCA score of the two groups (neg).

**FIGURE 2:**
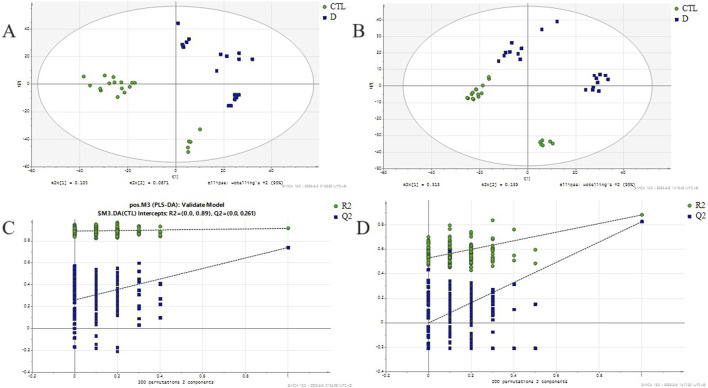
PLS-DA score plot and permutation validation for discriminating lung adenocarcinoma patients from healthy controls. **(A)** PLSDA score scatter plot between lung adenocarcinoma group and healthy control in positive ion mode (R^2^X = 0.262,R^2^Y = 0.99,Q2 = 0.945); **(B)** PLSDA score scatter plot between lung adenocarcinoma group and healthy control in negative ion mode(R^2^X = 0.563, R^2^Y = 0.896, Q2 = 0.743); **(C)** Permutation test evaluating the robustness of the PLS-DA model (n = 200 permutations, R2=(0.0,0.917), Q2=(0.0,0.018)) in positive ion mode; **(D)** Permutation test evaluating the robustness of the PLS-DA model (n = 200 permutations, R2=(0.0,0.658), Q2=(0.0,0.0676)) in negative ion mode; CTL - Healthy Control group, D - lung adenocarcinoma group.

A total ion flow chromatogram (TIC), as shown in Supplementary Figures S1A,B, was established to observe the retention time of the instrument, the stability of the instrument, and the amount of substances measured. The PCA analysis revealed the global metabolic variation between healthy controls (HC) and lung cancer, with the first two principal components(two components model: R2X[1] = 0.146, R2X[2] = 0.0752).NEG (two components model: R2X[1] = 0.396, R2X[2] = 0.0765)). The score plot demonstrated partial overlap between groups along PC1 but a discernible separation trend along PC2 (Figures 1A,B), suggesting underlying metabolic differences. Data preprocessing included log-transformation and unit variance scaling to mitigate dominance by high-abundance metabolites, with no outliers detected (Hotelling’s T^2^ < 95% confidence interval). To enhance group discrimination, Partial Least Squares-Discriminant Analysis (PLS-DA) was applied, yielding a model with two latent variables (R^2^X = 0.262, R^2^Y = 0.99) and robust predictive capability (Q^2^ = 0.945 via 7-fold cross-validation) (Figures 2A,B). Permutation testing (200 iterations) confirmed model validity, with a negative regression slope (p < 0.001), ruling out overfitting (Figures 2C,D).

### Differential metabolite screening

**FIGURE 3:**
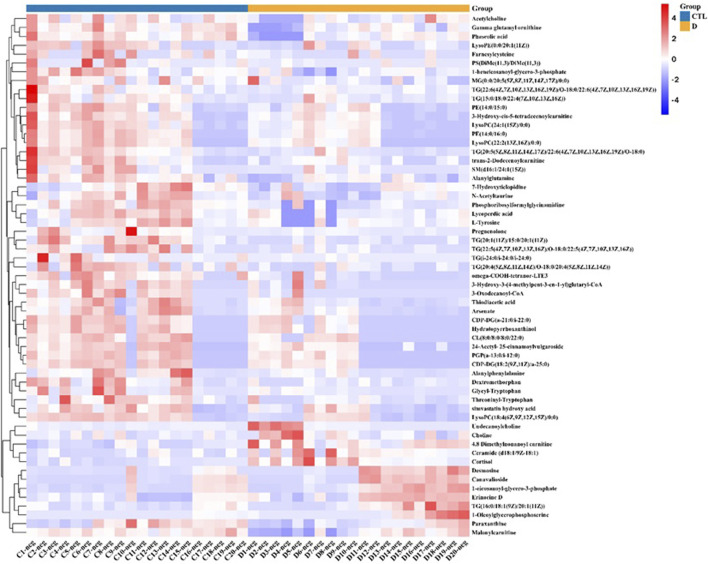
Volcano plot of differential metabolites, red dots represent significantly upregulated metabolites in the lung adenocarcinoma group, and green dots represent significantly downregulated metabolites in the lung adenocarcinoma group.

**FIGURE 4:**
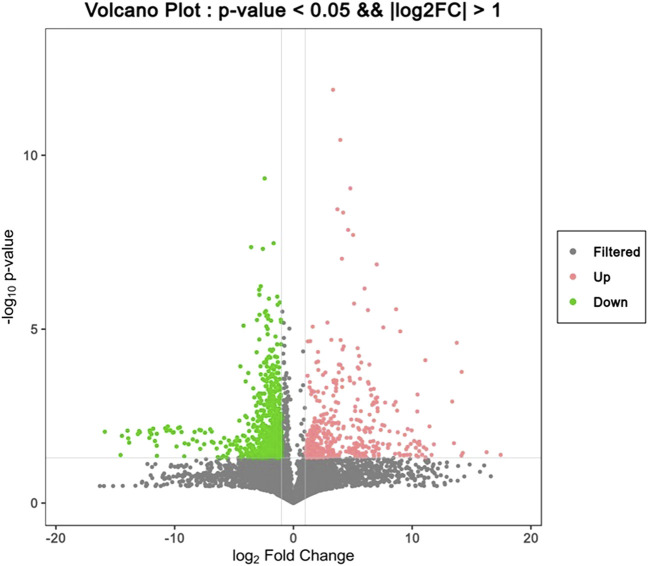
Heat map of differential metabolites, with red representing high content and blue representing low content.

To find the differential metabolites in the T1 lung adenocarcinoma group, variables with VIP greater than 1.0 were selected according to the PLS-DA model, Spss Statistics 18.0 combined with independent Analysis of Variance and T-test were applied. With P < 0.05 as statistical significance, Bonferroni correction was used for multiple test adjustments. Finally, 105 differential metabolites were identified, of which 82 were lipid metabolites, as shown in Supplementary Table S1. The volcanic and heatmap of the differential metabolites were obtained, as shown in Figures 3, Figures 4.

### Significant changes in differential metabolites of lung adenocarcinoma groups

In the condition of statistical significance (P < 0.05), the lung adenocarcinoma group was compared with the healthy control group. Fold change greater than 2 indicates a significant increase, and Fold change less than 0.5 indicates a significant decrease. 34 indicators were significantly increased, as shown in Supplementary Table S1. There are 71 indicators of significant downward adjustment, as shown in Supplementary Table S1.

### ROC curve analysis of differential metabolites

**FIGURE 5:**
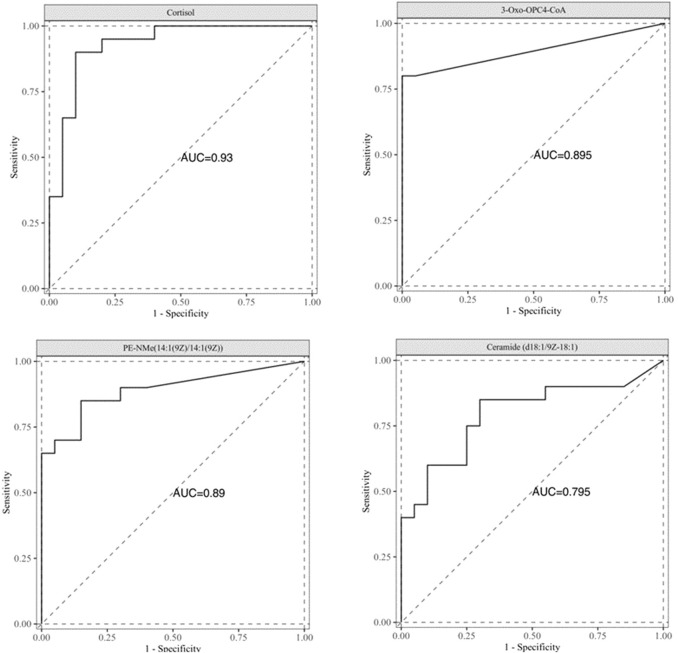
ROC curve analysis of differential metabolite.

In order to evaluate the differential metabolites found through multivariate statistical analysis, ROC curve analysis was performed for 105 differential metabolites, as shown in Supplementary Table S2. With AUC values 0.75 as the threshold, a total of four differential metabolites have been found, Cortisol, 3-Oxo-OPC4-CoA, PE-NMe(14:1(9Z)/14:1(9Z)), and Ceramide (d18:1/9Z-18:1), as shown in Figure 5. The AUC values were 0.930, 0.895, 0.890, and 0.795, respectively.

### Differential metabolite network analysis diagram

**FIGURE 6:**
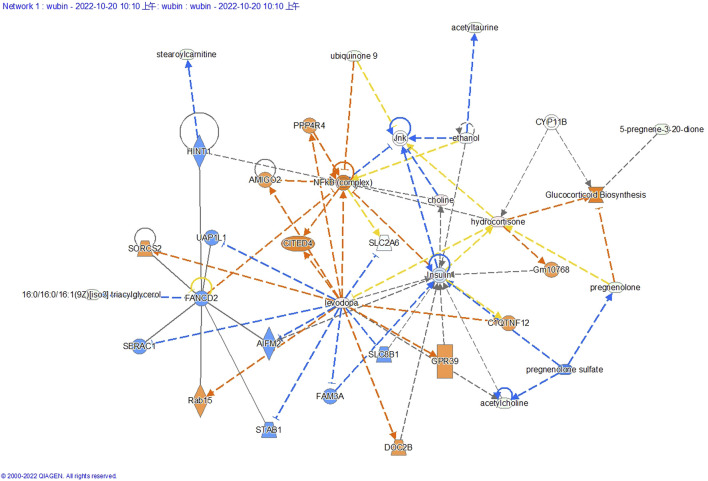
Differential metabolite-associated biological networks, typical pathways, and functions. In a network diagram, molecules are represented as nodes, and the biological relationship between two nodes is defined as a line. Orange symbols indicate upregulated metabolites, blue symbols indicate downregulated metabolites, yellow means found to be inconsistent with downstream molecular states, and gray means undetected. The solid line between molecules indicates the direct physical relationship between molecules, while the dashed line indicates the indirect functional relationship.

The interaction network generated through IPA analysis (Figure 6) integrates differentially expressed metabolites (fold change >2, FDR <0.05) and reveals critical metabolic reprogramming features associated with lung cancer progression. The figure presents a steroidal framework centered around 5-pregnen-3β-ol-20-one, characterized by its cyclopentanophenanthrene core with a hydroxyl group at the C3 position and a ketone moiety at C20. A dehydration reaction (−H_2_O) is indicated at the C3 hydroxyl, likely leading to the formation of a double bond, a common step in steroid biosynthesis to generate derivatives such as progesterone analogs. Subsequent modifications include the introduction of a fluorine atom (Flairine) at a strategic position, inferred to enhance metabolic stability and receptor interaction through its electronegative properties, and an acetyl group attached via esterification, potentially serving as a protective modification to modulate solubility or act as a prodrug mechanism. The sequential transformations—dehydration, halogenation, and acetylation—highlight a synthetic pathway aimed at optimizing pharmacokinetic properties, such as prolonged half-life and targeted bioactivity. Structural ambiguities, notably the incomplete closure of the upper-right ring system, are resolved through standard steroidal numbering conventions, while the absolute stereochemistry (e.g., β-configuration at C3) is presumed based on typical biosynthetic pathways.

## Discussion

Due to the influence of smoking, air pollution and the aging population, the incidence of lung adenocarcinoma is increasing yearly. However, the early clinical symptoms of lung adenocarcinoma patients are not obvious, and most patients are already in the local advanced stage or metastases when diagnosed, with a low 5-year survival rate and a very high recurrence rate (Ray et al., 2023). In recent years, researchers have explored the etiology and pathogenesis of lung adenocarcinoma from many angles. Still, so far, no theory can effectively explain the process of its occurrence and development. Although several blood-based tests have been developed to help classify lung cancer, non-invasive and reliable methods and biomarkers for early lung cancer detection are still lacking (Cohen et al., 2018). Presently, the specificity and sensitivity of commonly used diagnostic markers for lung adenocarcinoma in clinical practice are low, showing poor diagnostic value, and a single diagnostic marker is difficult to diagnose and classify a disease accurately. Therefore, the search for early warning markers of lung adenocarcinoma has become the top priority to improve the clinical diagnosis and treatment of lung adenocarcinoma, especially in stage T1, which is of great significance to improve the 5-year survival rate.

Our study identified four biomarkers-cortisol, 3-Oxo-OPC4-CoA, PE-NMe(14:1(9Z)/14:1(9Z)), and Ceramide (d18:1/9Z-18:1)-as potential diagnostic candidates for early-stage (T1) lung adenocarcinoma. These metabolites have not been specifically reported as biomarkers for T1-stage lung adenocarcinoma. In the context of T1 stage lung adenocarcinoma, understanding the role of them could provide insights into novel therapeutic strategies and prognostic markers. Cortisol, a glucocorticoid hormone produced by the adrenal glands, plays a significant role in various physiological processes, including metabolism, immune response, and stress regulation. While cortisol is extensively studied in adrenal pathologies and stress-related disorders, its role as a metabolic biomarker in cancer is less defined. In adrenocortical carcinoma (ACC), cortisol overproduction is a diagnostic and prognostic marker, with guidelines recommending its assessment in clinical management (Fassnacht et al., 2018). However, no direct evidence links cortisol to T1-stage lung adenocarcinoma. Its immunosuppressive effects and metabolic reprogramming properties (e.g., promoting glycolysis) suggest potential relevance in tumor microenvironments, but this remains unexplored in early lung cancer. 3-Oxo-OPC4-CoA, a key intermediate in fatty acid β-oxidation, reflects the reliance of cancer cells on altered lipid metabolism to fuel rapid proliferation, is not explicitly studied in T1-stage adenocarcinoma. However, fatty acid oxidation (FAO) is a recognized metabolic adaptation in cancer cells, including lung cancer, to support energy demands and survival. Compounds targeting FAO pathways (e.g., etomoxir) are under investigation, implying that intermediates like 3-Oxo-OPC4-CoA could serve as metabolic markers, though this requires validation (Fassnacht et al., 2018). PE-NMe(14:1(9Z)/14:1(9Z)), a phosphatidylethanolamine derivatives, is implicated in membrane dynamics and signaling across cancers. There was no studies specifically address PE-NMe species in lung adenocarcinoma. Their structural role in membrane fluidity and interaction with oncogenic signaling pathways suggests potential utility in early tumor detection, though this remains speculative. Ceramides, including Ceramide (d18:1/9Z-18:1), are sphingolipids that have been implicated in the regulation of cell death and survival. They are known to induce apoptosis in cancer cells, making them a potential target for cancer therapy (Ung et al., 2024).

There are 84 lipid metabolites among 105 differentially expressed metabolites in our study,it is important to underscores the critical role of lipid metabolism dysregulation in the pathogenesis of T1-stage lung adenocarcinoma. The differential lipid classes identified in our study—phosphatidylcholines (PC), phosphatidylethanolamines (PE), triglycerides (TG), and ceramides—highlight distinct metabolic disruptions linked to T1-stage lung adenocarcinoma development. Phosphatidylcholine (PC) is critical for membrane structure and signaling, with emerging roles in T1-stage lung adenocarcinoma. Lipidomic studies using LC-MS revealed distinct monounsaturated-to-saturated PC ratios between adenocarcinoma and normal tissues, suggesting diagnostic potential for histological subtypes (Muranishi et al., 2019). Genetic studies highlight glycerophosphocholine acyltransferase Gpc1’s role in PC remodeling, influencing cancer cell membrane dynamics through saturated/unsaturated PC balance (Anaokar et al., 2019). Advanced imaging techniques, particularly intra- and peritumoral radiomic features from CT scans, enhance subtype discrimination in pure ground-glass nodules (Jiang et al., 2024). Integrating lipid metabolism (PC profiling) and radiomics offers a multimodal strategy to refine early diagnosis and targeted therapies for T1-stage lung adenocarcinoma. Phosphatidyl ethanolamine (PE) is an abundant phospholipid in mammalian cells. It has significant activity and regulatory functions in cell proliferation, metabolism, organelles, entosis, autophagy, stress response, apoptosis, and senescence. Using ultra-high performance liquid chromatography-time-of-flight mass spectrometry, Chen et al. conducted non-targeted metabolomics analysis on the serum of patients with early lung cancer, patients with benign lung diseases and healthy controls and found that lipid metabolism profile of patients with early lung cancer had significant changes, among which phosphatidylcholine and phosphatidylethanolamine could be used as biomarkers of early NSCLC (Chen et al., 2018). Triglycerides, studied for their role in cancer metabolism, are linked to tumor progression in T1-stage lung adenocarcinoma. Inflammatory biomarkers like CXCL9, associated with improved disease-free and overall survival, suggest interconnected metabolic-inflammatory pathways influencing prognosis (Zhang et al., 2020). Surgical interventions, such as segmentectomy (lung-preserving) versus lobectomy, offer comparable oncological outcomes for T1 tumors, balancing function preservation and efficacy (Bilgi and Swanson, 2019; Hattori et al., 2016). Advanced imaging techniques, including CT habitat analysis, improve preoperative prediction of invasive patterns (e.g., spread through air spaces), guiding personalized management (Peng et al., 2024). Integrating metabolic insights, surgical strategies, and imaging innovations enables a multidisciplinary approach to optimize T1-stage adenocarcinoma treatment and patient outcomes. In order to describe the expression characteristics of abnormal lipid metabolism in early lung cancer, Wang et al. used high-performance liquid chromatography and mass spectrometry to conduct non-targeted lipid omics analysis and data analysis on the plasma of 171 patients with T1 lung cancer and 140 healthy adults. They identified 1478 metabolites of 14 types of lipids under the positive ion mode. In the negative ion mode, 708 metabolites of 13 kinds of lipids were found, and lysophosphatidylcholine, phosphatidylcholine, and triglyceride were identified as the most important characteristics of early cancer detection (Wang et al., 2022).

In addition to triglyceride metabolism, lipid changes are related to free fatty acid metabolism, sphingomyelin, and ceramide metabolism. Fatty acid metabolism is involved in tumorigenesis and development and plays an important role in many biological functions, such as cell membrane formation, energy storage, and molecular transmission of tumor signals (Ye and Li, 2023). Various bioactive intermediates produced during fatty acid metabolism regulate the occurrence and development of lung tumor cells through multiple steps. Highly active fatty acid metabolism provides favorable conditions and necessary energy for lung tumor cells’ survival, proliferation and invasion. Enzymes in the sphingomyelin metabolic pathway have been found to be dysfunctional in lung cancer, desensitizing cells to treatment response and promoting cell proliferation, migration, and angiogenesis by down-regulating ceramides levels (Janssen et al., 2020). Sphingomyelin is considered a sphingolipid, accounting for about 85% of all sphingolipids in cell membranes. Sphingomyelin consists of a phosphatidylcholine head, fatty acid tail, and sphingomyelin. The chemical structure of sphingomyelin gives it a significant role in signaling pathways. Sphingomyelin isolated from cancer cells can also promote growth, angiogenesis, and metastasis. Ceramide, as the center of sphingolipid metabolism and the precursor of complex sphingolipids, can be used to synthesize complex sphingolipids, including sphingolipids or glucose sphingolipids, and can also be degraded by ceramidase to produce sphingoside, which is mainly used to induce cell cycle arrest and apoptosis, and control the growth and spread of cancer cells (Gomez-Larrauri et al., 2021). Ceramide is widely recognized as a central tumor suppressor molecule due to its ability to induce apoptosis and anti-proliferative response, which can promote apoptosis in almost all cells, tissues and organs. High ceramide levels can directly or indirectly disrupt REDOX homeostasis, and increase membrane permeability and reactive oxygen species formation. Cang et al. conducted a non-targeted metabolomics study on 20 NSCLC patients and 10 healthy volunteers, collected positive and negative ion scanning data of subjects in the two groups. The results showed a large number of lipid changes between lung cancer and healthy group, such as phosphatidylcholine, phosphatidylethanolamine, lysophosphatidylcholine, lysophosphatidylethanolamine, sphingomyelin, ceramide, diglycerol, triglyceride and cholesterol (Cang et al., 2021). Mitchell et al. used non-targeted lipidomics to detect the difference in lipid profiles between cancerous and non-cancerous lung tissue samples from 86 patients with suspected stage I or IIA primary NSCLC. The results showed that compared with non-cancerous tissues, the lipid profiles of cancerous tissues were consistently higher in sterols, higher in sphingolipids and lower in cardiolipins. And there were significant lipid profile differences between cancer and non-cancer samples (Mitchell et al., 2021). Fan et al. conducted non-targeted lipidomics analysis of 67 NSCLC cases and 18 healthy subjects based on liquid chromatoclC-mass spectrometry, and the results showed that compared with remote non-cancerous tissues, the levels of phosphatidylethanolamine, lysophosphatidylglycerol, ceramide (Cer), phosphatidylcholine (PC), phosphatidylglycerol and triglyceride in lung cancer tissues were increased. Levels of free fatty acids, cardiolipin and phosphatidylinositol were decreased, and ceramide and diacylglycerol were used as combined tissue markers to distinguish adenocarcinoma from squamous cell carcinoma, PC(38:6) and Cer G3 (d18:1/22:0) were used as potential markers to distinguish lung cancer from healthy tissue. The sensitivity, specificity and area under the curve are (0.78, 0.70 and 0.78) and (0.94, 0.67 and 0.80), respectively (Fan et al., 2020).

As these examples suggest, a greater understanding of the differences in lipid metabolism between lung cancer groups and healthy controls is an essential first step in building more complete models of early progression and warning of lung cancer. To screen efficient and specific metabolic markers for the early diagnosis of T1 lung adenocarcinoma, this study adopted the metabolomics technology based on liquid chromatography and high-resolution mass spectrometry (LC-MS) to establish a complete set of methods, including sample pre-processing, data collection and data processing to explore the changes of serum lipid metabolites in T1 lung adenocarcinoma group and healthy control group. Differential metabolites were screened out, and the diagnostic value of potential differential metabolites was evaluated using ROC curve. The results showed that Cortisol, 3-Oxo-OPC4-CoA, PE-NMe(14:1(9Z)/14:1(9Z)), and Ceramide (d18:1/9Z-18:1) can be used as metabolic markers for the early diagnosis of T1 lung adenocarcinoma, which can be used for mass screening of high-risk populations for cancer prevention, and reduce unnecessary radiation exposure and invasive diagnostic procedures.

## Conclusion

To sum up, this study is based on the serum samples of the subjects, which is simple and less invasive, and easy to repeat the detection over time. The potentially confusing effects of lipid metabolism complications, such as diabetes, hyperlipidemia and atherosclerosis, were excluded. The changes of lipid metabolism in serum of patients with stage T1 lung adenocarcinoma were explored by combining metabolomics technology and multivariate statistical analysis. It was found that the serum metabolic profile of the lung adenocarcinoma group was significantly different from that of the healthy control group. Cortisol, 3-Oxo-OPC4-CoA, PE-NMe(14:1(9Z)/14:1(9Z)), and Ceramide (d18:1/9Z-18:1) can be used as combined lipid markers for the early diagnosis of T1 lung adenocarcinoma, improving the diagnostic efficiency of T1 lung adenocarcinoma. Of course, our study is also related to limitations; the relatively small study sample and regional data set may question the generality therefore, the clinical applicability of these combined markers will need to be confirmed by further large-scale cohort studies.

## Data Availability

The original contributions presented in the study are included in the article/Supplementary Material, further inquiries can be directed to the corresponding author.

## Supplementary material

SUPPLEMENTARY FIGURE S1
**(A)** The overlay of TIC diagram of the QC samples (pos) **(B)** The overlay of TIC diagram of the QC samples (neg).

## References

[B1] Adams S. J. Stone E. Baldwin D. R. Vliegenthart R. Lee P. Fintelmann F. J. (2023). Lung cancer screening. Lancet 401 (10374), 390–408. 10.1016/s0140-6736(22)01694-4 36563698

[B2] Anaokar S. Kodali R. Jonik B. Renne M. F. Brouwers J. Lager I. (2019). The glycerophosphocholine acyltransferase Gpc1 is part of a phosphatidylcholine (PC)-remodeling pathway that alters PC species in yeast. J. Biol. Chem. 294 (4), 1189–1201. 10.1074/jbc.RA118.005232 30514764 PMC6349126

[B3] Bilgi Z. Swanson S. J. (2019). Current indications and outcomes for thoracoscopic segmentectomy for early stage lung cancer. J. Thorac. Dis. 11 (Suppl. 13), S1662–s1669. 10.21037/jtd.2019.07.06 31516739 PMC6706620

[B4] Blandin Knight S. Crosbie P. A. Balata H. Chudziak J. Hussell T. Dive C. (2017). Progress and prospects of early detection in lung cancer. Open Biol. 7 (9), 170070. 10.1098/rsob.170070 28878044 PMC5627048

[B5] Buma A. I. G. Schuurbiers M. M. F. van Rossum H. H. van den Heuvel M. M. (2023). Clinical perspectives on serum tumor marker use in predicting prognosis and treatment response in advanced non-small cell lung cancer. Tumour Biol. 46, S207–S217. 10.3233/tub-220034 36710691

[B6] Cang S. Liu R. Jin W. Tang Q. Li W. Mu K. (2021). Integrated DIA proteomics and lipidomics analysis on non-small cell lung cancer patients with TCM syndromes. Chin. Med. 16 (1), 126. 10.1186/s13020-021-00535-x 34838074 PMC8627049

[B7] Cao W. Chen H. D. Yu Y. W. Li N. Chen W. Q. (2021). Changing profiles of cancer burden worldwide and in China: a secondary analysis of the global cancer statistics 2020. Chin. Med. J. Engl. 134 (7), 783–791. 10.1097/cm9.0000000000001474 33734139 PMC8104205

[B8] Chen P. Liu Y. Wen Y. Zhou C. (2022). Non-small cell lung cancer in China. Cancer Commun. (Lond) 42 (10), 937–970. 10.1002/cac2.12359 36075878 PMC9558689

[B9] Chen T. Zhang R. Zhang Y. Yuan W. (2019). Early potential metabolic biomarkers of primary postpartum haemorrhage based on serum metabolomics. Ginekol. Pol. 90 (10), 607–615. 10.5603/gp.2019.0105 31686419

[B10] Chen Y. Ma Z. Shen X. Li L. Zhong J. Min L. S. (2018). Serum lipidomics profiling to identify biomarkers for non-small cell lung cancer. Biomed. Res. Int. 2018, 5276240. 10.1155/2018/5276240 30175133 PMC6106807

[B11] Cohen J. D. Li L. Wang Y. Thoburn C. Afsari B. Danilova L. (2018). Detection and localization of surgically resectable cancers with a multi-analyte blood test. Science 359 (6378), 926–930. 10.1126/science.aar3247 29348365 PMC6080308

[B12] Fan Y. Noreldeen H. A. A. You L. Liu X. Pan X. Hou Z. (2020). Lipid alterations and subtyping maker discovery of lung cancer based on nontargeted tissue lipidomics using liquid chromatography-mass spectrometry. J. Pharm. Biomed. Anal. 190, 113520. 10.1016/j.jpba.2020.113520 32784094

[B13] Fassnacht M. Dekkers O. M. Else T. Baudin E. Berruti A. de Krijger R. (2018). European society of endocrinology clinical practice guidelines on the management of adrenocortical carcinoma in adults, in collaboration with the European network for the study of adrenal tumors. Eur. J. Endocrinol. 179 (4), g1–g46. 10.1530/eje-18-0608 30299884

[B14] Gomez-Larrauri A. Ouro A. Trueba M. Gomez-Muñoz A. (2021). Regulation of cell growth, survival and migration by ceramide 1-phosphate - implications in lung cancer progression and inflammation. Cell Signal 83, 109980. 10.1016/j.cellsig.2021.109980 33727076

[B15] Hattori A. Matsunaga T. Takamochi K. Oh S. Suzuki K. (2016). The oncological outcomes of segmentectomy in clinical-T1b lung adenocarcinoma with a solid-dominant appearance on thin-section computed tomography. Surg. Today 46 (8), 914–921. 10.1007/s00595-015-1256-6 26471507

[B16] Hirsch F. R. Scagliotti G. V. Mulshine J. L. Kwon R. Curran W. J. Jr. Wu Y. L. (2017). Lung cancer: current therapies and new targeted treatments. Lancet 389 (10066), 299–311. 10.1016/s0140-6736(16)30958-8 27574741

[B17] Huang C. Freter C. (2015). Lipid metabolism, apoptosis and cancer therapy. Int. J. Mol. Sci. 16 (1), 924–949. 10.3390/ijms16010924 25561239 PMC4307283

[B18] Janssen E. M. Dy S. M. Meara A. S. Kneuertz P. J. Presley C. J. Bridges J. F. P. (2020). Analysis of patient preferences in lung cancer - estimating acceptable tradeoffs between treatment benefit and side effects. Patient Prefer Adherence 14, 927–937. 10.2147/ppa.S235430 32581519 PMC7276327

[B19] Jiang W. Qu T. Liu W. Shi H. Zhang Y. (2024). Intra- and peritumoral-based radiomics for preoperatively assessing the pathological subtype of T1-stage lung adenocarcinoma presenting as pure ground-glass nodules. Technol. Cancer Res. Treat. 23, 15330338241305432. 10.1177/15330338241305432 39648728 PMC11626656

[B20] Johnson C. H. Ivanisevic J. Siuzdak G. (2016). Metabolomics: beyond biomarkers and towards mechanisms. Nat. Rev. Mol. Cell Biol. 17 (7), 451–459. 10.1038/nrm.2016.25 26979502 PMC5729912

[B21] Li R. Z. Fan X. X. Duan F. G. Jiang Z. B. Pan H. D. Luo L. X. (2018). Proscillaridin A induces apoptosis and suppresses non-small-cell lung cancer tumor growth via calcium-induced DR4 upregulation. Cell Death Dis. 9 (6), 696. 10.1038/s41419-018-0733-4 29899551 PMC5999972

[B22] Lin L. An L. Chen H. Feng L. Lu M. Liu Y. (2021). Integrated network pharmacology and lipidomics to reveal the inhibitory effect of qingfei oral liquid on excessive autophagy in RSV-induced lung inflammation. Front. Pharmacol. 12, 777689. 10.3389/fphar.2021.777689 34925035 PMC8672039

[B23] Long J. Zhang C. J. Zhu N. Du K. Yin Y. F. Tan X. (2018). Lipid metabolism and carcinogenesis, cancer development. Am. J. Cancer Res. 8 (5), 778–791. 29888102 PMC5992506

[B24] Mayers J. R. Wu C. Clish C. B. Kraft P. Torrence M. E. Fiske B. P. (2014). Elevation of circulating branched-chain amino acids is an early event in human pancreatic adenocarcinoma development. Nat. Med. 20 (10), 1193–1198. 10.1038/nm.3686 25261994 PMC4191991

[B25] Mitchell J. M. Flight R. M. Moseley H. N. B. (2021). Untargeted lipidomics of non-small cell lung carcinoma demonstrates differentially abundant lipid classes in cancer vs. Non-cancer tissue. Metabolites 11 (11), 740. 10.3390/metabo11110740 34822397 PMC8622625

[B26] Muranishi Y. Sato T. Ito S. Satoh J. Yoshizawa A. Tamari S. (2019). The Ratios of monounsaturated to saturated phosphatidylcholines in lung adenocarcinoma microenvironment analyzed by Liquid Chromatography-Mass spectrometry and imaging Mass spectrometry. Sci. Rep. 9 (1), 8916. 10.1038/s41598-019-45506-3 31222099 PMC6586780

[B27] Nooreldeen R. Bach H. (2021). Current and future development in lung cancer diagnosis. Int. J. Mol. Sci. 22 (16), 8661. 10.3390/ijms22168661 34445366 PMC8395394

[B28] Patti G. J. Yanes O. Siuzdak G. (2012). Innovation: metabolomics: the apogee of the omics trilogy. Nat. Rev. Mol. Cell Biol. 13 (4), 263–269. 10.1038/nrm3314 22436749 PMC3682684

[B29] Peng X. Zhao H. Wu S. Jia D. Hu M. Guo B. (2024). Habitat-based CT radiomics enhances the ability to predict spread through air spaces in stage T1 invasive lung adenocarcinoma. Front. Oncol. 14, 1436189. 10.3389/fonc.2024.1436189 39464700 PMC11502297

[B30] Ray M. A. Akinbobola O. Fehnel C. Saulsberry A. Dortch K. Wolf B. (2023). Surgeon quality and patient survival after resection for non-small-cell lung cancer. J. Clin. Oncol. 41 (20), 3616–3628. 10.1200/jco.22.01971 37267506 PMC10325770

[B31] Sung H. Ferlay J. Siegel R. L. Laversanne M. Soerjomataram I. Jemal A. (2021). Global cancer statistics 2020: GLOBOCAN estimates of incidence and mortality worldwide for 36 cancers in 185 countries. CA Cancer J. Clin. 71 (3), 209–249. 10.3322/caac.21660 33538338

[B32] Ung J. Kassai M. Tan S. F. Loughran T. P. Jr. Feith D. J. Cabot M. C. (2024). The drug transporter P-glycoprotein and its impact on ceramide metabolism-an unconventional ally in cancer treatment. Int. J. Mol. Sci. 25 (18), 9825. 10.3390/ijms25189825 39337312 PMC11432138

[B33] Wang G. Qiu M. Xing X. Zhou J. Yao H. Li M. (2022). Lung cancer scRNA-seq and lipidomics reveal aberrant lipid metabolism for early-stage diagnosis. Sci. Transl. Med. 14 (630), eabk2756. 10.1126/scitranslmed.abk2756 35108060

[B34] Wishart D. S. (2016). Emerging applications of metabolomics in drug discovery and precision medicine. Nat. Rev. Drug Discov. 15 (7), 473–484. 10.1038/nrd.2016.32 26965202

[B35] Ye W. Li X. (2023). Development of fatty acid metabolism-related models in lung adenocarcinomaA Review. Med. Baltim. 102 (1), e32542. 10.1097/md.0000000000032542 PMC9829279 36607846

[B36] Zhang Y. Sun B. Hu M. Lou Y. Lu J. Zhang X. (2020). CXCL9 as a prognostic inflammatory marker in early-stage lung adenocarcinoma patients. Front. Oncol. 10, 1049. 10.3389/fonc.2020.01049 32714866 PMC7347039

[B37] Zhu Z. Zhang L. Lv J. Liu X. Wang X. (2020). Trans-omic profiling between clinical phenoms and lipidomes among patients with different subtypes of lung cancer. Clin. Transl. Med. 10 (4), e151. 10.1002/ctm2.151 32898330 PMC7438979

