# Supplementary figures and images for: Early potential metabolic biomarkers of T1 stage lung adenocarcinoma based on serum metabolomics
Source: Front Mol Biosci. 2025 Apr 25;12:1544774. doi: 10.3389/fmolb.2025.1544774 (PMC12061714; doi:10.3389/fmolb.2025.1544774)

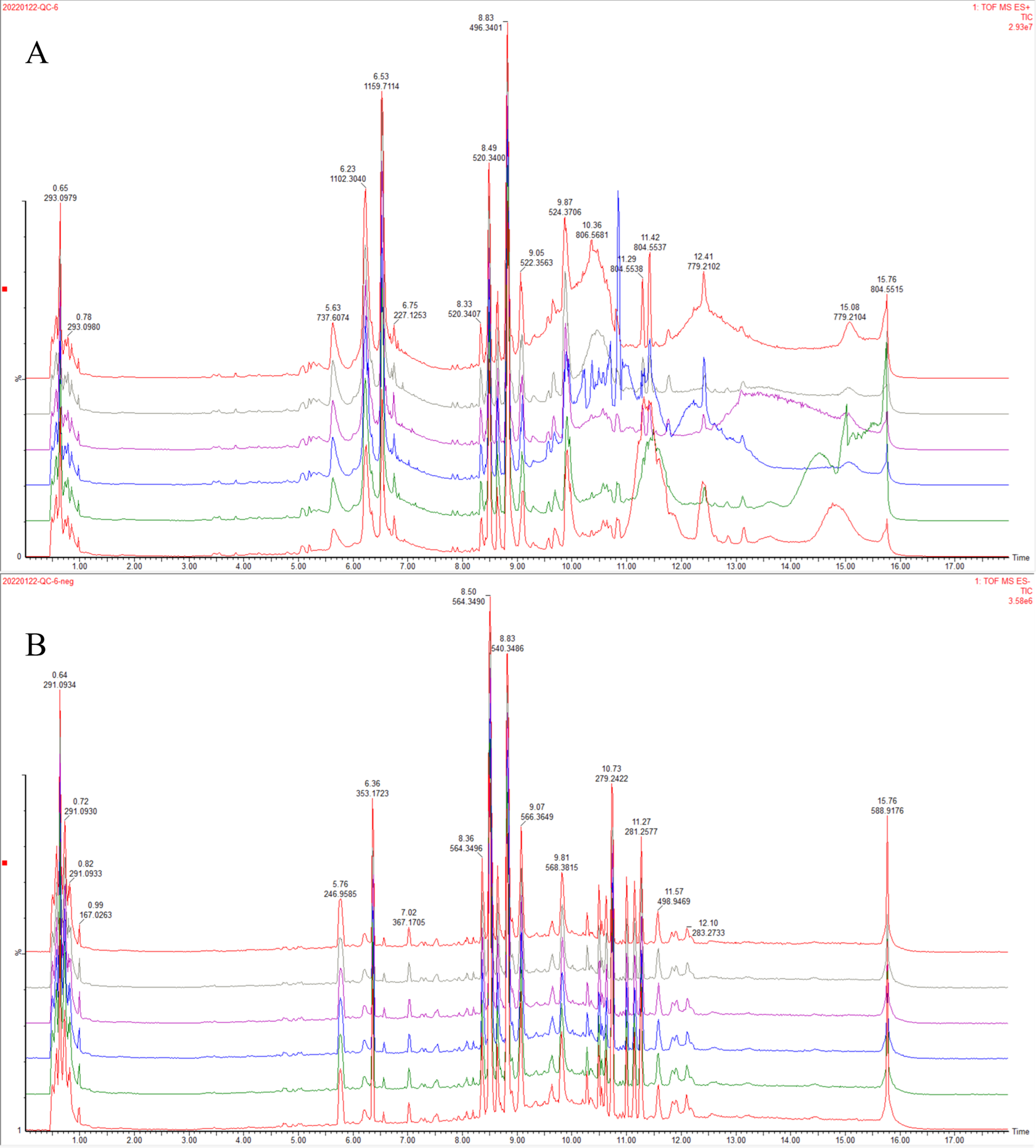

Supplement: Supplementary file 1 [file Image1.tif]
